# Vaccines for prion diseases: a realistic goal?

**DOI:** 10.1007/s00441-023-03749-7

**Published:** 2023-02-11

**Authors:** Scott Napper, Hermann M. Schatzl

**Affiliations:** 1 Vaccine and Infectious Disease Organization, University of Saskatchewan, Saskatoon, SK Canada; 2 Department of Biochemistry, Microbiology and Immunology, University of Saskatchewan, Saskatoon, SK Canada; 3 Calgary Prion Research Unit, Faculty of Veterinary Medicine, University of Calgary, Calgary, AB Canada

**Keywords:** Prion, Chronic wasting disease, Vaccine, Immunotherapy, Wildlife vaccine

## Abstract

Prion diseases are fatal infectious neurodegenerative disorders and prototypic conformational diseases, caused by the conformational conversion of the normal cellular prion protein (PrP^C^) into the pathological PrP^Sc^ isoform. Examples are scrapie in sheep and goat, bovine spongiform encephalopathy (BSE) in cattle, chronic wasting disease (CWD) in cervids, and Creutzfeldt–Jacob disease (CJD) in humans. There are no therapies available, and animal prion diseases like BSE and CWD can negatively affect the economy, ecology, animal health, and possibly human health. BSE is a confirmed threat to human health, and mounting evidence supports the zoonotic potential of CWD. CWD is continuously expanding in North America in numbers and distribution and was recently identified in Scandinavian countries. CWD is the only prion disease occurring both in wild and farmed animals, which, together with extensive shedding of infectivity into the environment, impedes containment strategies. There is currently a strong push to develop vaccines against CWD, including ones that can be used in wildlife. The immune system does not develop a bona fide immune response against prion infection, as PrP^C^ and PrP^Sc^ share an identical protein primary structure, and prions seem not to represent a trigger for immune responses. This asks for alternative vaccine strategies, which focus on PrP^C^-directed self-antibodies or exposure of disease-specific structures and epitopes. Several groups have established a proof-of-concept that such vaccine candidates can induce some levels of protective immunity in cervid and rodent models without inducing unwanted side effects. This review will highlight the most recent developments and discuss progress and challenges remaining.

## Introduction

### Prion mechanism

Prion diseases are fatal transmissible spongiform encephalopathies (TSEs) in human and animals that are characterized by spongiform degeneration and progressive neuronal loss in the central nervous system (CNS) (Prusiner 1982, 1998; Wadsworth and Collinge 2011). These diseases are caused by the accumulation of the pathological isoform (PrP^Sc^) of the cellular prion protein (PrP^C^) (Aguzzi et al. 2008; Budka 2003; Prusiner 1982; Prusiner et al. 1998; Tatzelt and Schatzl 2007; Wadsworth and Collinge 2011). Prion diseases are prototypic conformational disorders, diseases where a normal cellular host protein changes its conformation in a way which usually results in gain of a toxic function. How exactly PrP^c^ is converted into PrP^Sc^ is not fully understood at the molecular level, and it depends on whether the manifestation is acquired by infection, genetic, or sporadic routes (Aguzzi et al. 2008; Budka 2003; Prusiner 1982; Prusiner et al. 1998; Tatzelt and Schatzl, 2007; Wadsworth and Collinge 2011). Most experimental evidence points to a molecular mechanism where seeds of PrP^Sc^ incorporate PrP^C^ molecules in a seeded aggregation process (Jarrett and Lansbury 1993; DeMarco and Daggett 2004; Igel-Egalon et al. 2019). The generation of initial seeds depends on the manifestation form, with intra-molecular conformational changes in PrP preceding inter-molecular ones for sporadic prion diseases (DeMarco and Daggett 2004; Igel-Egalon et al. 2019; Taguchi et al. 2018). In genetic forms, the underlying amino acid exchange seems to initiate and/or accelerate this mechanism. For acquired prion diseases, PrP^Sc^ seeds are provided by the invading prions. In all three situations, the existing cellular clearance mechanisms dealing with aggregated proteins are bypassed or overwhelmed (Schatzl 2013; Marrero-Winkens et al. 2020).

When a certain threshold of PrP^Sc^ seeds is exceeded, PrP^Sc^ propagates in an autocatalytic process that increases the infectious load. Although this is a post-translational process that is strictly dependent on the availability of PrP^c^ substrate (Bueler et al. 1993; Mallucci et al. 2003), it is not fully understood whether post-translational modifications in PrP isoforms are causally involved in this process (Aguilar-Calvo et al. 2021; Makarava and Baskakov 2022). The infectious character of prion diseases is reflected at the cellular level by transfer of prion infectivity from cell to cell, inside and outside of the CNS, which results in the infection of naïve cells. Since cell culture models exist that recapitulate acute and persistent prion infection, the cell biology of intracellular generation of prions, interaction with cellular pathways and clearance mechanisms, and infection of new cells are rather well studied and subject of many review articles (Heiseke et al. 2010; Priola 2017; Krance et al. 2020; Heumüller et al. 2022).

At the organism level, the manifestation of disease depends on where infection is initiated. For sporadic and genetic prion diseases, which represent the majority of human prion diseases, this seems to be the CNS, in an endogenous mechanism that does not involve infection from outside. This is different for acquired forms of disease, the ones usually found in animal prion diseases, which clearly involve an exogenous process of infection. For the latter, the route of infection decides on what cell types and tissues are initially involved. The most relevant route of infection for acquired prion diseases of man and animals is the oral one: prions are ingested and get access to lymphatic and nervous tissues that line the digestive tract. This process is well studied in rodent models or the relevant large animal models of prion infection, and many mechanistic insights were obtained using transgenic and knock-out mouse models (Prusiner et al. 1998; Weissmann et al. 2001; Groschup and Buschmann 2008; Wadsworth et al. 2010; Moreno and Telling 2017). Depending on the species and route of infection, propagation of prions involves lymphatic tissues and parts of the peripheral nervous system (PNS). From cranial nerves, there is a direct entry into the CNS from lymphatic tissues and secondary lymphoid organs (Bartz et al. 2005; Shearin and Bessen 2014; Beekes 2021). Prions can be detected in the blood with highly sensitive methods, but the extent to which prionemia plays a pathogenetic role in peripheral prion infection is unclear (Elder et al. 2015).

Similarly, there is an anterograde transport of prion infectivity from the infected CNS to the periphery, likely also from extra-CNS sites of prion propagation, which can result in prion shedding into body fluids and excretions and result in lateral infection from individual to individual (Saunders et al. 2008; Bessen et al. 2010; Haley et al. 2011). The type of prion disease and the species involved mostly dictate whether such a scenario can result in population-level relevant scenarios, with epidemics as most extreme situation. Most human prion disease patients are not contagious on a daily level to their fellow human beings, although certain medical procedures and devices can transmit prions, in particular when they had access to infected CNS (Brown et al. 2000). Similarly, BSE is not contagious from cow to cow under normal circumstances but can be transmitted between cattle by prion contaminated feed sources. Here, infection was caused by prion-tainted animal feed, most likely meat and bone meal (Kimberlin 1992; Wells and Wilesmith 1995). The potential for lateral infection is high for scrapie in sheep and chronic wasting disease (CWD) in cervids (Dickinson et al. 1974; Williams and Miller 2002; Mathiason et al. 2006). Besides such intra-species infection, selected prion diseases have the potential to infect other species, with BSE as most prominent example (Hill et al. 1997; Bruce et al. 2007). Although many species can be infected experimentally when using the intracerebral route of infection, there are natural limitations summarized under the concept of “species barrier” in prion disease research (Scott et al. 1989; Hill and Collinge 2001; Béringue et al. 2008). The molecular and cellular determinants of this species barrier are not fully understood, but it is likely that regional homologies or non-homologies in the tertiary structure of invading PrP^Sc^ and PrP of the host species play a key role in this scenario (Prusiner et al. 1990; Collinge and Clarke 2007; Sigurdson et al. 2010).

Although prions can replicate in a variety of cell types and tissues outside the CNS, clinical disease manifestation is unique to the CNS, mainly because of progressive loss of neurons. Additional pathological hallmarks are vacuolation, reactive astrocytosis, and deposition of extracellular proteinaceous plaques consisting of PrP^Sc^ (Kovács et al. 2004). The type of prions influences these hallmarks, and a variety of different pathological signatures in the CNS can exist within a given species. In analogy to genetic variants as known for microbes, the concept of “prion strains” was introduced to describe this phenomenon (Bessen and Marsh 1992; Carlson et al. 1994; Bessen et al. 1995; Weissmann 2009). Since prions do not contain encoding nucleic acid and strictly use PrP^C^ of the host species for their replication, conformational variability at the PrP level and existence of PrP^Sc^ conformers is widely accepted as the underlying molecular mechanism (Bessen and Marsh 1992; Carlson et al. 1994; Bessen et al. 1995; Weissmann 2009; Collinge 2010; Carta and Aguzzi 2022). Molecular signatures that discriminate prion strains can be clinical symptoms, type of vacuolation, and PrP^Sc^ plaque distribution in the brain, immunoblot profile, and resistance to denaturing agents and proteinase K (DeArmond et al. 1994; Aguzzi et al. 2007; Wadsworth and Collinge 2011). Similar as various RNA viruses exist as a dynamic quasi-species population in the host, a probably more restricted quasi-species nature of PrP^Sc^ conformers is in place. The dynamic nature of these quasi-species conformers was evident in experimental drug treatment studies and indicates a selection process at the structural level (Li et al. 2010; Berry et al. 2013; Bian et al. 2014).

### Prion-like diseases

There is a growing number of human neurodegenerative diseases which implicate a prion-like mechanism (Meyer-Luehmann et al. 2006; Eisele et al. 2010; Goedert et al. 2010; Jucker and Walker 2013; Ayers and Cashman 2018; Ayers et al. 2020; Kim et al. 2022). Whereas in prion diseases a bona vide infectious agent (prion) propagates and spreads within and between individuals, a prion-like mechanism is limited to the spread of disease-causing protein aggregates from cell to cell within a host. This concept was pioneered in mouse models in which disease was induced, or “seeded,” by experimental injection of brain homogenates from patients (Meyer-Luehmann et al. 2006; Eisele et al. 2010). This started with transgenic mouse models of Alzheimer’s disease (AD) and a focus on a-beta and was extended to a variety of other proteopathies (Meyer-Luehmann et al. 2006; Eisele et al. 2010; Goedert et al. 2010; Jucker and Walker 2013; Ayers and Cashman 2018; Ayers et al. 2020; Kim et al. 2022). The immediate implications of these experiments were emphasized by the transmission of disease in Parkinson’s disease (PD) patients that had received brain transplants, implicating transmission of α-synuclein misfolding from host to transplant (Li et al. 2008).

The family of prion-like proteins is growing and includes now a-beta (AD), tau (tauopathies), α-synuclein (PD), and SOD in amyotrophic lateral sclerosis (ALS) and often using the term prion for these proteins (e.g., tau prions) (Marciniuk et al. 2013). This “transmissibility” of the misfolded form of these proteins has been recapitulated at the cellular level in cell culture models, initially for a-syn and tau aggregates, and model prions (Ren et al. 2009; Frost et al. 2009; Krammer et al. 2009; Hofmann et al. 2013). The combination of in vivo and in vitro studies provides solid evidence for cellular uptake, release, and transport of protein seeds along neural pathways and networks. Another important similarity with prions is the existence of prion-like strains, which affect selective vulnerability and targeting in the brain and thereby influence the neuropathological and clinical attributes of disease.

While the risks of nosocomial transmission of the prion-like diseases are quite low, emerging evidence implicates situations which could lead to the de novo initiation of these diseases. For PD, disease progression seems to follow anatomical patterns and involve prion-like propagation events (Hawkes et al. 2009). The Braak hypothesis also proposes that PD pathogenesis starts outside the brain, triggered by exogenous insults in the gut and olfactory system (Hawkes et al. 2009; Braak and Del Tredici 2016), a scenario with striking similarities to peripheral prion infection. Others have suggested that bacterial amyloids may be responsible for initiating a variety of protein-misfolding neurodegenerative diseases, “mapranosis” for microbiota-associated proteopathy, and neuroinflammation (Friedland and Chapman 2017).

## Prion diseases of different species

### Human

Human prion diseases are very rare, fatal neurodegenerative disorders that include Creutzfeldt–Jakob disease (CJD), Gerstmann–Sträussler–Scheinker syndrome (GSS), fatal familial insomnia (FFI), kuru, variant CJD (vCJD), and variably protease-sensitive prionopathy (VPSPr) (Goldfarb et al. 1994; Ghetti et al. 1995; Gambetti et al. 1995; Gajdusek 2008; Capellari et al. 2011; Notari et al. 2018). All three manifestations are found — sporadic, familial, and acquired by infection (which includes iatrogenic transmission and via consumption of prion-infected foodstuffs) — although the sporadic form of CJD is the most common, with an incidence of one case per million people per year worldwide. CJD is not contagious in normal social interactions; however, various scenarios for iatrogenic transmissions have been described, involving dura mater and cornea transplants, growth hormone therapies, and EEG electrodes (Brown et al. 2000). Sporadic CJD commonly becomes symptomatic after the age of 50–55 years. From the first symptoms to the fatal outcome, the disease lasts only a few months, with usually longer clinical progression for the other human prion diseases.

vCJD appeared in the UK for the first time in 1996. More than 230 cases have been recorded, the vast majority of them the UK, with France coming next (Diack et al. 2014). The disease showed up in several European countries, Japan, Saudi Arabia, Taiwan, Canada, and the USA. The majority of these cases are probably “imported” ones, as the patients are thought to have been infected in the UK. At least four cases of secondary vCJD have occurred through blood transfusions or factor VIII preparations since 2004 (Llewelyn et al. 2004). It is currently unknown how many people in the UK might be infected with vCJD. Archived surgical materials like the tonsil and appendix were analyzed for markers of PrP^Sc^ to get an estimate on the level of occult infections in the UK population to the conclusion that 0.1–1.0% of that at risk population might be asymptomatically infected (Hill et al. 1999). Whether these individuals would ever develop clinical disease would likely depend on a combination of aspects of the infectious dose (physical properties and dose) as well as genetic susceptibilities of the host. Defined single-nucleotide polymorphisms of the human prion gene have been shown to impact susceptibility to vCJD (Saba and Booth 2013) and kuru (Lee et al. 2001).

The incubation period of human prion diseases ranges from a minimum of 4 years (some kuru and iatrogenic CJD cases) to more than 50 years. The incubation period of vCJD is at least 10 years, and periods of 20–30 years are considered probable. In the genetic forms, the incubation period usually lasts four to five decades, providing a theoretical window of opportunity for treatment modalities. Unfortunately, only few potential anti-prion compounds were tested in clinical trials, with very limited success (Zafar et al. 2019). Active vaccination is currently not considered an option for human prion diseases, given the very rare incidence rates. The situation might be different for familial prion diseases which can be diagnosed long before clinical symptoms start. An experimental therapy with a monoclonal antibody was recently described for a small number of CJD patients (Mead et al. 2022). The treatment was able to penetrate the CNS without inducing neurological side effects, warranting the need for future formal clinical trials. Such a treatment could be also an option for post-exposure prophylaxis when the time of potential infection is known, for example, laboratory accidents.

### Animal

Animal prion diseases are usually acquired by infection and can be associated with significant lateral transmission (Pattison 1970; Hunter 1991; Wells and Wilesmith 1995; Requena et al. 2016; Williams and Young 1980; Babelhadj et al. 2018). Food animal species like sheep, goat, deer, elk, camel, and cattle are affected. Prion infection in such species can have pronounced economic impacts, affecting the entire economies, consumer behaviors, and international trade and involving extensive active and passive surveillance mechanisms. The extreme example here is BSE in its epidemic form that devastated entire markets and cost billions of dollars in lost revenue and extra costs in many countries. Interestingly, prion diseases in sheep, goat, and cervids provided important insights into the role of amino acid polymorphisms in the prion protein, as a disease-modifying factor influencing susceptibility or providing a relative resistance. Many of the widely used prion strains for experimental research are derived from scrapie isolates, which had been passaged and adapted to mice and murine cells (e.g., RML, 22L, and Me7 prion strains). Finally, it was research in scrapie and then BSE that fuelled the concept of atypical prion diseases, which most likely have a sporadic origin and are not the result of infection from an external source (Benestad et al. 2008; Boujon et al. 2016).

### Scrapie

has been described in small ruminants like sheep and goats (Pattison 1970; Hadlow 1999). Descriptions go back to the early eighteenth century, and linguistic research suggests that the disease was already known in ancient times. The disease has different names in various countries, referring to the two characteristic symptoms of the disease: itching and ataxia. For scrapie, groundbreaking work helped to define the concept of prion strains and the role of polymorphisms in PrP. As a result of the latter, sheep populations have been selected that were considered resistant against classical scrapie. Scrapie is spread worldwide, with exception of Australia and New Zealand. Scrapie is transmitted both vertically and horizontally, and transmission is promoted by direct, close contact between animals. The high stability of scrapie prions in the environment explains why the disease can recur in farms in which no sheep were kept for a year or longer after culling. Scrapie has an extensive involvement of the lymphatic system, and prion infectivity is found in the spleen, lymph nodes, small intestine, and tonsils. Scrapie is most often managed through culling of affected animals and herds, together with active and passive surveillance. Such efforts are complicated by the existence of atypical scrapie which means a high probability of ongoing cases despite any implemented control measures (Acín et al. 2021). To date, there is no evidence that scrapie represents a zoonotic threat. Vaccination or therapy is generally not considered of importance for scrapie.

### BSE

in its epidemic form was described for the first time in the UK in 1986 and has led to enormous financial losses for agriculture sectors and entire economies in several countries (Wells and Wilesmith 1995; Requena et al. 2016). This also started a critical discussion on the limitations of an “industrialization of agriculture,” first in Europe and then throughout the world. Upon the first descriptions in cows with a CNS disorder, an epidemic developed in the UK that reported up to 3500 new clinical cases per month at its peak in 1992. Since the mid-1980s, more than 184,000 clinical cases have been recorded, affecting over 50% of UK cattle farms. However, the real significance of BSE might be its zoonotic potential, as over 230 cases of BSE in humans (vCJD) have been described in England, France, and several other countries (Diack et al. 2014; Llewelyn et al. 2004). BSE has been detected almost worldwide, including countries like Canada, Israel, Oman, Japan, and the USA. In Europe, Ireland, Portugal, France, Spain, Switzerland, and Germany have exhibited the most detections of BSE besides the UK. BSE emerged due to repetitive feeding of scrapie- or BSE-contaminated meat and bone meal, the latter considered more likely as a source given the existence of atypical BSE. Two control measures by the British government are important to mention. In 1988, a feeding ban was imposed on meat and bone meal for ruminants. This action effectively broke the infection chain, although evident only after of 3–5 years, the incubation period of BSE in cattle. Horizontal transmission plays no significant role in cattle, and BSE has no marked lymphotropism in bovines. Second, specified risk materials (SRM) from cattle were no longer allowed to enter the human food chain, starting in 1989. SRM include all parts of the CNS, the spine, and some visceral organs. This step dramatically reduced the exposure of the human population. In recent years, atypical BSE has been found in several countries worldwide, mostly in older cows and at a very low frequency. It comes in at least two forms and has altered histopathological and biophysical properties, such as a reduced proteinase K resistance (Casalone et al. 2004; Dudas and Czub 2017). Therefore, it could be overlooked with the usual tests for BSE. Although considered a sporadic form in etiology, one form of atypical BSE is clearly infectious in animal experiments, and zoonotic potential cannot be fully excluded at present time (Dudas and Czub 2017). For containment of epidemic BSE, bans on high-risk feed materials, culling, and surveillance strategies were very effective. Therapeutic or vaccination approaches are therefore not considered necessary.

### CWD

has been detected in cervid species in North America, South Korea, and Scandinavia and poses a serious threat to animal health (Williams 2005; Gilch et al. 2011; Saunders et al. 2012; Benestad and Telling 2018). CWD is responsible for cervid population declines and has an adverse economic impact on cervid hunting and tourism industries (DeVivo et al. 2017; Hannaoui et al. 2017). CWD is considered the most contagious prion disease, and substantial shedding of CWD prion infectivity into the environment via urine, feces, and saliva significantly contributes to disease spreading (Tamgüney et al. 2009a, b; Nichols et al. 2009; John et al. 2013). The long-term perseverance of CWD infectivity in environment reservoirs, including soil, water, and plants, makes disease management very challenging (Pritzkow et al. 2015). Whether CWD transmits naturally to other animal species or humans is a matter of concern that needs continued investigation. CWD was experimentally transmitted to cattle, pigs, cats, hamsters, and bank voles (Hamir et al. 2011; Mathiason et al. 2013; Di Bari et al. 2013; Moore et al. 2017). This raises the question whether the range of natural hosts of CWD prions can and will extend beyond cervids. Of particular importance is livestock that shares pastures contaminated with CWD prions. CWD prions would thereby indirectly have access to the human food chain. CWD transmission studies in transgenic mouse models expressing PrPs from various species including ovine, bovine, and human showed low or absent ability of CWD prions to cross relevant species barriers (Tamgüney et al. 2009b; Wilson et al. 2012; Kurt et al. 2015; Wadsworth et al. 2022). Interestingly, a more recent study found atypical disease and fecal prion shedding in transgenic mice expressing human PrP when infected with deer prions, indicating zoonotic potential of CWD (Hannaoui et al. 2022). In addition, transmission of CWD into non-human primates via the oral route (Marsh et al. 2005; Race et al. 2009, 2018) and efficient in vitro conversion of human PrP by CWD prions (Barria et al. 2011; Wang et al. 2021) should also not be ignored. Additionally, the existence of various CWD prion strains combined with the known PrP polymorphisms generates a dynamic, emerging, and complex scenario for future CWD transmission risks.

### Is there an expanding threat?

The escalating threat posed by CWD includes a rather uncontrolled geographic expansion within cervids, uptake by plants and preservation in the environment by soils, unpredictable evolution of CWD prions, and possibly some zoonotic potential (Williams 2005; Gilch et al. 2011; Saunders et al. 2012; Benestad and Telling 2018). To put this into numbers, the US Fish and Wildlife Service estimates that in 2016 alone, 9 million Americans — roughly 1 in 36 — pursued big game such as deer and elk with ~ $26 billion spent on hunting. US and Canadian government agencies have collectively invested billions of dollars to manage CWD in free-ranging and farmed cervid populations, with little success so far. Elk, deer, and reindeer farming in Canada have emerged as an alternative livestock industry, and wild-living deer, elk, moose, and caribou are important economic drivers in Canada to attract tourists and hunters. There are around 55,000 farmed and more than 2 million wild cervids in Canada. CWD has significantly impaired the Canadian deer and elk farming industry when the first case of CWD was found in a deer farm in 1996. CWD will cost the Canadian cervid industry many millions for double fencing to separate free-ranging from captive deer and up to half a billion dollars to close CWD-infected cervid farms. Testing and disposal of carcasses and carcass parts are additional costs associated with the disease. Native cervid herds with high CWD prevalence are showing population declines (DeVivo et al. 2017; Hannaoui et al. 2017), threatening a robust hunting industry and wildlife conservation efforts. Regarding human health, it is estimated that between 7000 and 15,000 CWD-infected cervids are consumed in North America by humans annually, with an increase of ~ 20% per year (Geist et al. 2017). CWD testing depends on jurisdiction and is not mandatory in all hunting units. The annual prevalence in Alberta, measured by testing for prions in hunted heads, steadily increased from < 1% in 2005 to > 15% in mule deer and 5% in white-tailed deer (WTD). Long considered a North American problem, CWD showed up more recently in three Scandinavian countries, Norway, Sweden, and Finland (Benestad et al. 2016; Benestad and Telling 2018). Comparative transmission studies in transgenic mice and bank voles so far indicate that North American and Scandinavian CWD isolates are different, basically excluding import from the USA and Canada as a source of the European infections (Tranulis et al. 2021; Bian et al. 2021). There are also gross differences between the CWD strains in the three affected species (wild reindeer, red deer, and moose), and some are discussed as examples for a sporadic origin, not acquired by infection, resembling atypical scrapie and atypical BSE. Depopulation was mostly used as a containment strategy in Norway, and it has yet to be seen how effective this will be.

### Is there a need for new tools?

Containment strategies in wild cervids like depopulation and selective harvesting were not effective in stopping the expansion of CWD in North America. This asks for new tools, and active vaccination of wild cervids is now widely considered a promising strategy. Although there was initially skepticism whether vaccines can work for prion diseases in general, there is now solid experimental evidence of the feasibility of this approach. In contrast to prion diseases in other species, CWD is not controllable through routine animal management practices as wild-living animals are affected. This represents the next challenge, it must be a vaccine that works in a wildlife scenario. Even if active vaccination alone might not be able to fully protect cervids against CWD, it can be the central element of concerted containment strategies, aiming to reduce disease in animals, shedding of prions, and prion load in cervids and environment. CWD likely needs a One Health approach, and vaccination will be a perfect example to address health in animals, humans, and the environment.

### Opportunities and challenges to developing prion vaccines

Historically, vaccines have been the most effective tactic for the management of infectious diseases in humans and animals. There is a clear need, and emerging optimism, for the potential to develop vaccines for prion diseases. Efforts to develop vaccines for other proteinopathies are showing encouraging progress, and relative to those diseases, the prion diseases have the advantage because they present a clearly defined, cell surface-accessible, immunotherapeutic target. Extensive investigations have demonstrated the ability of PrP-reactive antibodies to impair prion propagation in vitro as well as for passive and active immunization to delay disease progression in animal models. While encouraging, development of an effective prion vaccine remains an elusive goal that is challenged by several unique aspects of prion biology, including overcoming immunological tolerance, concerns of the safety of inducing immune responses to a self-protein, uncertainties of the mechanisms of immune protection, and establishing benchmarks of success of prion vaccines which may be unique for human and animal applications.

### Challenges to developing a prion vaccine (self-tolerance)

For traditional infectious diseases, a distinct boundary exists between the host and infectious agent. That line is blurred in the prion diseases, even to the immune system. The ability of the immune system to protect the host depends on its remarkable capacity to induce a virtually unlimited range of highly specific responses, all the while avoiding reactivities with self-molecules. This unresponsiveness of the immune system to self-molecules is referred to as immune tolerance. PrP^C^ falls within the realm of immune tolerance, and, as the transition to PrP^Sc^ does not involve changes to the polypeptide sequence, this immune privilege also extends to the pathological isoform. With that, most prion infections proceed to their fatal outcomes in the absence of an immune response (Porter et al. 1973; Kasper et al. 1982). While anti-PrP antibodies have been detected in the end stages of disease (Sassa et al. 2010), more typically, the immune system does not perceive, nor respond to, PrP^Sc^ as an infectious threat. Thus, the unique biology of prions shelters the infectious agent from immune activation, allowing unchecked progression of the disease and complicating the development of immunotherapies; overcoming immune tolerance to PrP is a central obstacle to the development of prion vaccines (Mabbott 2015).

Mechanistically, immune tolerance reflects the active depletion of those T and B lymphocytes whose receptors show reactivity to self-molecules (Zinkernagel et al. 2001) as well as active suppression of immune responses to self-molecules by Treg cells (Sakaguchi et al. 2008). The extent to which self-tolerance restricts immune responses to PrP is demonstrated by the ease of induction of PrP-reactive antibodies in PrP ^–/–^ mice (Kascsak et al. 1987; Williamson et al. 1996; Krasemann et al. 1996) as well as xenogenic systems (Rubenstein et al. 1999). In contrast, PrP vaccines that are not optimized for immunogenicity typically achieve only modest titers of low-affinity antibodies in wild-type animals (Paramithiotis et al. 2003).

Immunological tolerance to PrP can be overcome through recapitulation of the missing immune components; this includes the introduction of either PrP-sensitized CD4 + T cells from PrP^–/–^ donors (Gourdain et al. 2009) or transgenic T cells with a PrP-reactive T cell receptor (Iken et al. 2011). Similarly, administration of dendritic cells loaded with PrP peptides can result in protective immune responses (Bachy et al. 2010). While these studies highlight the potential to achieve immune activation to PrP, as well as associated degrees of protection, these approaches are inconsistent with real-world vaccines for either human or animals. Human prion diseases are an insufficient threat to justify vaccination of the general population. Instead, human prion immunotherapies would most commonly be initiated at the onset of clinical symptoms or to individuals with genetic predisposition to disease. The former is problematic as the clinical symptoms depend on disease progression to the CNS where opportunities for immunotherapy are limited. For animal prion diseases, due to restrictions of acceptable cost and intensiveness of administration, efforts to overcome immune tolerance are confined to more traditional approaches of vaccinology, including selection and optimization of vaccine targets (antigens, also called immunogens) as well as strategies of formulation and delivery.

### Overcoming self-tolerance (antigen selection and optimization)

Prion vaccines can be broadly categorized as either subunit or peptide, depending on whether the vaccine antigen represents the entirety, domain, or a specific region of the PrP polypeptide. Each category of prion vaccine antigen presents distinct opportunities for overcoming immune tolerance.

For prion subunit vaccines, one approach to overcome immune tolerance is to use heterologous versions of PrP with species-specific sequence variations. For example, while mouse PrP was immunologically tolerated in BALB/c mice (Polymenidou et al. 2004), both bovine and sheep PrP were highly immunogenic (Ishibashi et al. 2007). A challenge to this approach is ensuring that the induced antibodies are reactive to the PrP isoforms of the host and/or infecting prion species as in some instances the antibodies induced by either recombinant or heterologous PrP antigens are unreactive with PrP^C^ or PrP^Sc^ (Heppner and Aguzzi 2004). Presentation of the PrP antigen as recombinant dimers can also overcome immune tolerance, even to homologous sequences (Gilch et al. 2003; Abdelaziz et al. 2017). Absorption of PrP to Dynabeads has also been employed as a delivery vehicle for prion subunit vaccines (Tayebi et al. 2009). Others have overcome self-tolerance through DNA vaccines that drive the expression of PrP that is coupled to either stimulatory T cell epitopes (Alexandrenne et al. 2010), carrier proteins that promote antigen uptake and MHC class I presentation (Han et al. 2011), or lysosomal targeting signal peptides (Fernandez-Borges et al. 2006).

Peptide-based prion vaccines allow for prioritization of specific regions of PrP. This specificity, however, is often at the further expense of immunogenicity as short peptides, especially those of self-proteins, are often weakly immunogenic. A vaccine based on a peptide target of PrP induced only weak IgM responses, even when coupled to an immunogenic carrier and formulated with harsh adjuvants (Paramithiotis et al. 2003). One approach to increase the immunogenicity of self-peptides is to increase their length through inclusion of additional, naturally occurring residues that flank the region of interest; the immunogenicity of the tripeptide TyrTyrArg (YYR) was increased by four orders of magnitude through the inclusion of additional residues on the N and C terminal sides of the core tripeptide (Hedlin et al. 2010). The relationship between peptide length and immunogenicity is not, however, absolute; additional residues can even reduce immunogenicity (Hedlin et al. 2010). Instead, the occurrence of B cell epitopes within the sequence better predicts immunogenicity. Software that forecasts the immunogenicity of regions of proteins based on endogenous B cell epitopes enables optimization of peptide epitope targets for their rapid translation into vaccines (Marciniuk et al. 2015).

Less conventional approaches of antigen selection have been taken to overcome immunological tolerance to PrP. Through a bioinformatic approach, Ishibashi et al. identified a non-mammalian protein, succinylarginine dihydrolase, that shared sequence similarity to those recognized by anti-PrP antibodies. Immunization of mice with this bacterial mimitope induced a PrP-specific antibody response with a modest degree of protection (Ishibashi et al. 2011). Wille and colleagues also employed a mimitope approach by introducing key residues of PrP into an immunogenic protein scaffold that enables the induction of conformation-specific immune responses against PrP^Sc^ (Fleming et al. 2022).

### Overcoming self-tolerance (vaccine formulation and delivery)

Following selection and optimization of a suitable antigen, there are additional parameters that can be utilized to overcome immune tolerance and facilitate vaccine delivery, including carrier proteins, adjuvants, and biological vectors.

### Carrier proteins

Rationale selection and optimization of antigens are often insufficient to overcome immune tolerance. These targets, especially peptide epitopes, must often be presented in the context of an immunogenic carrier proteins to elicit the T cell help required for strong immune humoral responses and efficient IgM to IgG class switching. Numerous carrier proteins have been investigated for prion vaccines including leukotoxin (Lkt) protein of Mannheimia haemolytica (Hedlin et al. 2010), rabies glycoprotein G (gG) (Taschuk 2017), blue carrier protein (Pilon et al. 2007), cholera toxin (Bade et al. 2006), heat-labile enterotoxin B subunit (Yamanaka et al. 2006), and heat shock proteins (Koller et al. 2002). While few investigations have considered attributes of the same prion epitope in the context of different carriers, there is evidence that carrier selection influences the magnitude, duration, and nature of the induced immune response (Taschuk et al. 2015a, b). Further, certain carriers, such as cholera toxin and *Escherichia coli* heat-labile enterotoxin, seem better suited for mucosal vaccines (Bade et al. 2006; Sakaguchi and Arakawa 2007). Consideration is also required of whether peptide epitopes are added to the carrier through recombinant fusion or chemical conjugation. Expression as recombinant fusions is advantaged in terms of cost and consistency of epitope presentation, while chemical conjugation is often better suited for high-throughput screening of both carriers and peptides. In selecting a carrier, it is also important to consider compatibility with higher-order delivery vectors. For example, the ease of expression of Lkt recombinant fusions in bacteria, with subsequent purification as inclusion bodies, is well-suited to generate antigens for injected vaccines, but its poor expression in eukaryotic cells prohibits its use in viral vectored vaccines.

### Adjuvants

Vaccine adjuvants are another important variable in overcoming immune tolerance to PrP. Numerous adjuvants have been investigated for prion vaccines including Emulsigen plus (Hedlin et al. 2010), CpG (Rosset et al. 2004), complete Freund’s adjuvant (Tal et al. 2003), and Adjuvac™ (Pilon et al. 2007). The success of these adjuvants is often measured in their ability to induce high-titer antibody responses, which seems an appropriate metric of success of vaccines that prioritize humoral responses. It is, however, also necessary to define the extent to which the adjuvant impacts prion disease progression; adjuvant, Adjuvac^™^, accelerated prion disease (Pilon et al. 2007), while the adjuvant-only control of Freund’s complete adjuvant delayed prion disease (Tal et al. 2003). Finally, the ease to which that adjuvant can be translated to licensed humans or animal vaccines, from both financial and regulatory perspectives, is an important longer-term consideration of adjuvant selection.

### Biological vectors

The incorporation of PrP antigens into biological vectors can help overcome immune tolerance. A panel of hamster polyomavirus virus-like particles (VLPs) representing various PrP peptides induced epitope-specific immune responses, some of which offered protection from prion infection (Eiden et al. 2021). Others have used VLP based on bovine papillomavirus (BPV-1) in which a peptide sequence from murine PrP was inserted into a major capsid protein. This vector induced epitope-specific antibody responses that inhibited the replication of PrP^Sc^ in a tissue–culture model (Handisurya et al. 2007). VLPs based on murine leukemia virus (MLV), engineered to target a C terminal region of PrP, induced PrP^C^ reactive antibodies (Nikles et al. 2005). Parental administration of adenoviral vectors encoding a xenogenic PrP resulted in only marginal immunity against endogenous mPrP, although a moderate prolongation of survival was achieved (Rosset et al. 2009). Oral administration of a prion vaccine based on a replication-deficient human adenovirus induced both peripheral and mucosal epitope-specific immune responses (Taschuk et al. 2017). Mucosal delivery of PrP epitopes within *Salmonella*-based vectors induced protective responses in mice and cervids (Goni et al. 2005, 2008, 2015).

### Challenges to developing a prion vaccine (safety)

Safety, a primary consideration of any vaccine, takes on even greater significance for the protein-misfolding diseases (proteinopathies) due to recognition that the induction of immune responses towards self-proteins can have pathological consequences. Most infamously, a subset of patients involved a clinical vaccine trial for Alzheimer’s disease developed encephalitis (Nicoll et al. 2003). Within the proteinopathies, these safety concerns become even more complex and nuanced for the prion diseases. It is complex due to evidence that lymphoid tissues offer a more permissive environment for prion amplification (Beringue et al. 2012), such that vaccine-associated immune responses could traffic the misfolded species to regions that promote prion amplification to accelerate disease. It is nuanced in that prion vaccines that prioritize either the PrP^C^ or PrP^Sc^ isoforms are associated with unique safety risks.

### Potential dangers of reactivity to PrP^C^

While developing vaccines that target the healthy isoform of PrP may seem counterintuitive, two characteristics of PrP-/- animals support the philosophy and safety of this approach. Firstly, PrP-/- animals are impervious to prion infection, highlighting the absolute requirement of PrP^C^ for disease propagation (Bueler et al. 1993). Secondly, the absence of a severe loss-of-function phenotype with genetic ablation of PrP supports the safety of immunological depletion of this protein (Bueler et al. 1992). This must, however, be balanced with the appreciation that PrP^C^ reactive antibodies could have gain of function rather than loss-of-function, consequences. Supportive of this, the presence of PrP^C^ reactive antibodies, or their Fab fragments, in the brain resulted in neuronal apoptosis (Solforosi et al. 2004; Lefebvre-Roque et al. 2007) although subsequent investigations challenge this result (Klohn et al. 2012). PrP^C^ reactive antibodies have also been shown to result in inappropriate cell signal events (Cashman et al. 1990; Mouillet-Richard et al. 2000; Arsenault et al. 2012), superoxide-mediated cytotoxicity (Sonati et al. 2013), and stimulation of suppressor T cell lymphocytes (Isaacs et al. 2006).

These concerns of the safety of PrP^C^-specific vaccines must be balanced with the appreciation that have numerous vaccine trials have utilized this approach with no reports of significant pathologies. While this is reassuring, it must be noted that most of these efforts were focused on vaccine efficacy rather than safety. Further, there may not be a singular answer to the question of the safety of targeting PrP^C^ as antibodies with reactivities to different regions of PrP have unique pathological consequences; antibodies to the octarepeat are well tolerated, while those against the folded globular domain associate with neurotoxicity (Sonati et al. 2013). That it is possible to map the pathology-associated regions of PrP^C^, as well as those associated with neutralizing epitopes, offers the potential to generate peptide-based vaccines based on dual consideration of safety and efficacy (Reimann et al. 2016).

### Potential dangers of reactivity to PrP^Sc^

Targeting the misfolded species, whose presence is uniquely dependent on prion infection, appears a logical strategy to mitigate the safety concerns associated with auto-reactive antibodies. However, the events involved in the template-driven misfolding of PrP^C^ to PrP^Sc^ are not clearly understood, and there is the theoretical potential that PrP^Sc^-reactive antibodies could serve as chaperones that promote, or stabilize, misfolding intermediates, which could paradoxically lead to the induction of prion disease in otherwise healthy subjects.

Thus far, the concern that PrP^Sc^-specific vaccines could initiate prion disease have not been supported by experimental data. Antibodies to a region of PrP whose surface exposure was unique to misfolding did enhance the presentation of these regions but did not generate PrP^Sc^ (Paramithiotis et al. 2003). Similarly, prolonged incubation of polyclonal PrP^Sc^-reactive antibodies with brain homogenates failed to generate protease-resistant isoforms (Marciniuk et al. 2013). Finally, that induction of high-titer PrP^Sc^-specific antibodies in tg20 mice, which are genetically sensitized to prion disease, did not result in clinical nor biochemical indications of prion disease after 250 days ( Määttänen et al. 2013). While acceleration of disease was observed in elk receiving a PrP^Sc^-specific vaccination, it is uncertain of the extent to which the specificity of the vaccine contributed to that outcome (Wood et al. 2018).

While the absence of pathological consequences following prolonged incubation of PrP^Sc^-reactive antibodies in tg20 is reassuring, it should be noted that the sensitivity of these mice to prion disease is a consequence of overexpression of wild-type PrP rather than the introduction of PrP sequence variants with elevated risk for misfolding. The ability for PrP^Sc^-specific antibodies to promote misfolding may be conditional of the presence of misfolding prone PrP variants. Supportive of this, PrP^Sc^-specific antibodies reacted with a PrP variant associated with early onset familial dementia, but not wild-type PrP, in nanopore and immunoprecipitation experiments (Madampage et al. 2013). The reactivity of polyclonal PrP^Sc^-specific antibodies with this variant indicates the occurrence, and recognition, of subtle conformational differences and/or partially unfolded species. Prolonged incubation of the PrP^Sc^-specific antibodies with this misfolding prone PrP failed to generate protease-resistant PrP in vitro, although the extent to which these interactions could promote formation of PrP^Sc^ in vivo is uncertain. The potential for PrP^Sc^-induced misfolding of naturally occurring PrP variants will be of greater significance should these vaccines advance to application to outbred populations.

### Challenges to developing a prion vaccine (achieving protective immune responses)

The uncertainties regarding the mechanisms by which induced immune responses impact prion disease —initiation, progression, and pathology — is another challenge to the development of effective vaccines. It is difficult to develop and optimize vaccines in the absence of knowledge of the key determinants of protection. This includes consideration of humoral vs cellular responses and, within humoral responses, the relative importance of mucosal versus peripheral immunity. This situation is further complicated by species-specific differences of “what success look like” for an effective prion vaccine.

### Systemic vs mucosal responses

**Fig. 1:**
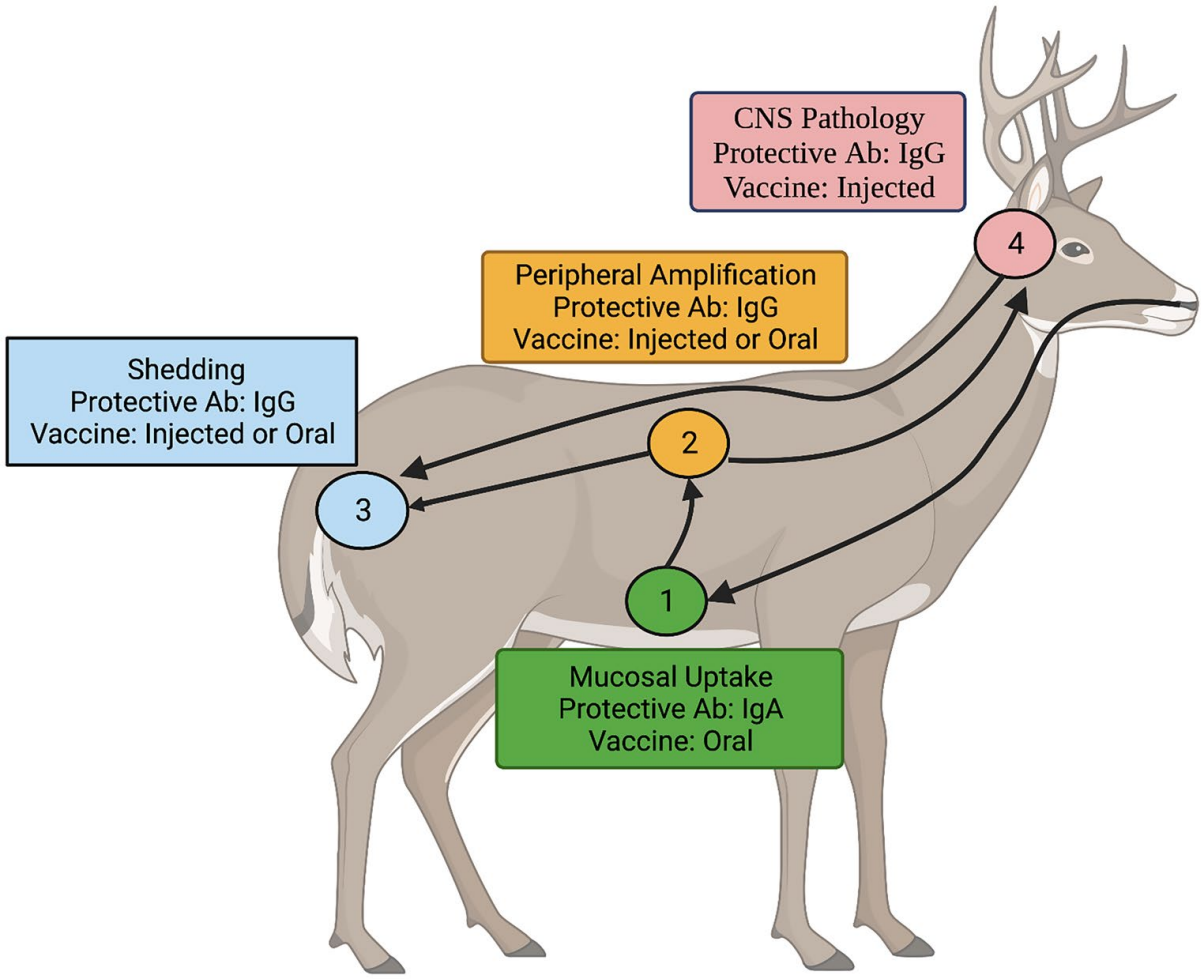
Stages of progression of CWD and potential points of immunotherapeutic intervention. (1) Mucosal uptake. Following oral ingestion, environmental prions are taken up through mucosal surfaces. Induction of IgA antibodies through oral vaccines offers the best chance to block uptake. (2) Peripheral amplification. Following uptake, prions undergo a stage of peripheral amplification. Induction of IgG antibodies, through either oral or injected vaccines can inhibit this process. (3) Shedding. Prions generated in periphery and CNS of the infected host are shed in urine and feces. IgG antibodies, induced through injected or oral vaccines, may restrict prion amplification to reduce shedding. (4) CNS pathology. After peripheral amplification, prions migrate to the CNS where they exert pathological consequences. While the BBB limits access of antibodies to the CNS, IgG antibodies, induced through either oral or injected vaccination, may minimize pathology

Depending on the source of infection, prion diseases can involve up to three stages: uptake at mucosal surfaces, peripheral amplification, and transmission to the CNS. Each stage presents unique opportunities and obstacles for immunotherapeutic intervention (Fig. 1).

For CWD, infection typically initiates following oral ingestion of prions with subsequent uptake at mucosal surfaces (Miller and Williams 2003; Beekes and McBride 2007). Consistent with the philosophy that the most effective way to deal with an infectious disease is to prevent it, blocking the uptake of gut-associated prions through induction of mucosal IgA responses may represent a critical line of defense (Fig. 1). This seems particularly true as thus far immunotherapy has only been able to delay, rather than abolish, disease progression once prions reach the periphery. It is noteworthy that strong peripheral responses can offer degrees of protection to peripheral challenge but are often less effective in protecting from oral exposure (Pilon et al. 2013). The most efficient protection from oral challenge likely depends on induction of mucosal IgA responses.

Achieving high titers of IgA antibodies is heavily dependent on the route of vaccine administration; parenterally administered vaccines induce serum IgG antibodies with muted IgA responses. A parenterally delivered prion vaccine favored IgG over IgA epitope-specific antibodies by an order of magnitude but with no significant correlation between serum IgG and mucosal IgA epitope-specific titers (Hedlin et al. 2010). Oral vaccination often results in a more balanced IgA to IgG profile (DeMagistris 2006); deer receiving an orally administered prion vaccine based on a human adenovirus delivery vector had an equalized titer and kinetics of responses of serum IgG to fecal IgA epitope-specific immunoglobins (Taschuk et al. 2017). Similarly, mucosal delivery of prion vaccines based on bacterial vectors induced both epitope-specific IgG and IgA responses (Goni et al. 2005, 2008, 2015) as did mucosal delivery of PrP epitopes conjugated to carrier proteins selected for their ability to induce mucosal responses (Yamanaka et al. 2006; Sakaguchi and Arakawa 2007).

The ability to induce both systemic and mucosal immune responses may be critical for an effective prion vaccine as the presence of both high-titer IgG and IgA antibodies, as compared to either high IgG or IgA antibodies alone, offered the greatest protection in a mouse oral infection model (Goni et al. 2008). Mucosal vaccination also delayed disease onset in an oral challenge model of cervids (Goni et al. 2015). Given these promising findings, and that oral immunization is the only viable option for wildlife vaccination, it is critical to explore the risks and benefits of orally administered prion vaccines. Most optimistically, IgA antibodies at the mucosal surface can prevent the uptake of consumed prions thereby preventing infection; in a worst-case scenario, the presence of PrP^Sc^-reactive mucosal immunoglobins could theoretically enhance the uptake of infectious particles from the gut to promote disease, as known as antibody-dependent enhancement (Xu et al. 2021).

In humans, other than the historic examples of kuru because of cannibalistic funeral practices or variant CJD resulting from consumption of prion-infected beef (Hill et al. 1997), most prion diseases originate in the periphery through either spontaneous or iatrogenic origins rather than initiating at mucosal surfaces (Will et al. 1998; Zou et al. 2008). Extensive trials utilizing parenterally administered prion vaccines support the ability of peripheral immune responses to slow the onset of clinical symptoms of disease. In these scenarios, systemic IgG responses likely restrict, but not eliminate, peripheral amplification of infectious particles. Following a period of peripheral amplification, which can last from weeks to decades depending on the species and nature of the challenge dose, prion disease migrate to the CNS where they exert their pathological consequences. Delaying the onset of symptoms of disease by minimizing peripheral amplification is certainly of importance in human patients, and minimizing the infectious load generated could be valuable to break the cycle of transmission in animals (Fig. 1).

Once the infectious agent reaches the CNS, the options for immunotherapy are limited by the relative impermeability of the blood–brain barrier (BBB) to immunoglobins (Neuwelt et al. 2011). Penetration of IgG immunoglobins across the BBB is quite limited (Podusio et al. 2001) resulting in concentrations of IgG in CSF that are approximately 500- to 1000-fold lower than in serum (Katsinelos et al. 2019). Consistent with this, an investigation of a parenterally administered prion vaccine found that the titers of epitope-specific antibodies were approximately three orders of magnitude lower than in CSF than in serum with strong correlation between the serum and CSF epitope-specific titers (Hedlin et al. 2010). Therefore, if limited to conventional vaccinology, achieving high-serum antibody titers may be critical to limit prion-induced neurodegeneration. There have also been efforts to translocate PrP-reactive immune responses past the BBB; camelid single-domain PrP-specific antibodies can cross the blood–brain barrier (David et al. 2014). Expression of a PrP-reactive antibody single-chain Fv fragment was achieved using a brain-engraftable microglial cell line with modest benefits on disease pathogenesis (Fujita et al. 2011). Further, vectored delivery of prion-specific single-chain fragment prolonged the survival time of prion-infected mice and decreased PrP^Sc^ in the brain (Wuertzer et al. 2008; Moda et al. 2012).

### Achieving protective immune responses (Th1 vs Th2)

Immunotherapy for prion diseases is typically based on the assumption that PrP-reactive immunoglobins offer therapeutic benefit through either destruction of the infectious isoform, neutralization of PrP^Sc^ to prevent further misfolding, or depletion of PrP^C^ to eliminate the substrate required for disease propagation, or a combination of these factors. This goes back to early studies in prion-infected cultured cells and in transgenic mice that showed that antibodies to PrP can block prion infection (Enari et al. 2001; Peretz et al. 2001; Heppner et al. 2001; Gilch et al. 2003). Regardless of the mechanism, these outcomes all depend on Th2 humoral responses. The protective value of anti-PrP antibodies is supported by the degrees of protection achieved through passive immunization of PrP-reactive antibodies as well as the correlation between the magnitude of humoral immune responses and the extent of protection (White et al. 2003; Bachy et al. 2010; Goni et al. 2008). While cellular immunity is generally not considered a significant contributor to protection for prion disease, vaccine-induced antigen-specific CD4 and CD8 cells have been described (Kaiser-Schulz et al. 2007). It is noteworthy that the transfer of either PrP-sensitized CD4 + T cells from PrP–/– donors (Gourdain et al. 2009) or transgenic T cells with a PrP-specific T cell receptor (Iken et al. 2011) slowed disease progression in the absence of induced antibody production. Similarly, that an adjuvant-only control was found to delay prion disease further indicates a potential more generalized immune activation to limit prion disease (Tal et al. 2003).

## Prion vaccine components

### Prion vaccine components (antigens)

The antigens of prion vaccines can be conceptually divided into three categories: those design to elicit antibodies which are specific for the PrP^C^, those prioritize response to the misfolded PrP^Sc^ isoforms, and those which generate antibodies that do not discriminate the PrP conformations.

### PrP^C^ as target

For traditional infectious diseases, the vaccine antigen(s) represent the entirety, or select biomolecules, of the invading pathogen, and therefore recognized as “foreign” by the host immune system. This is different for prion diseases. Only in the very early phases and if invading prions are from a different species are some PrP epitopes potentially recognized as “foreign.” This changes when prions start to replicate in the host, as this process entirely depends on recruitment of endogenous PrP^C^, which is a self-antigen, into newly produced PrP^Sc^. This explains why prion infections are not accompanied by a bona fide and detectable immune response against prions (Aucouturier and Carnaud 2002; Aguzzi 2003; Zabel and Avery 2015; Mabbott et al. 2018). T cells and most B cells are restricted to presentation of linear peptide epitopes on MHC molecules of antigen-presenting cells to their B and T cell receptors. Even more, isotype switching, somatic hypermutation, and terminal differentiation into highly specific (and long-living) plasma cells need help by antigen-specific CD4 cells. This all explains why the immune system seems to be “blind” for prions, although the role of microglia in the CNS is still a matter of debate (Perry 2004). Targeting PrP^C^ in active immunization is therefore complicated by the necessity to overcome self-tolerance against PrP.

A second problem is the risk to induce thereby undesirable side effects, both from over-reaching immune reactions and compromising the normal function of PrP^C^. For the latter, PrP^C^ is widely accepted as an adequate target for anti-prion activities, as severe loss-of-function phenotypes were not found in animal models (Bueler et al. 1993; Mallucci et al. 2003; Nicoll and Collinge 2009). Overall, there is in the meantime solid proof-of-concept that active immunization can break the self-tolerance against PrP to produce self-antibodies, without inducing unwanted side effects in experimental animal models (Abdelaziz et al. 2017; Eiden et al. 2021; Fernandez-Borges et al. 2006; Goni et al. 2005, 2008, 2015; Ishibashi et al. 2007; Heppner et al. 2001; Polymenidou et al. 2004; Rosset et al. 2009; Taschuk et al. 2015a, b, 2017).

**Fig. 2:**
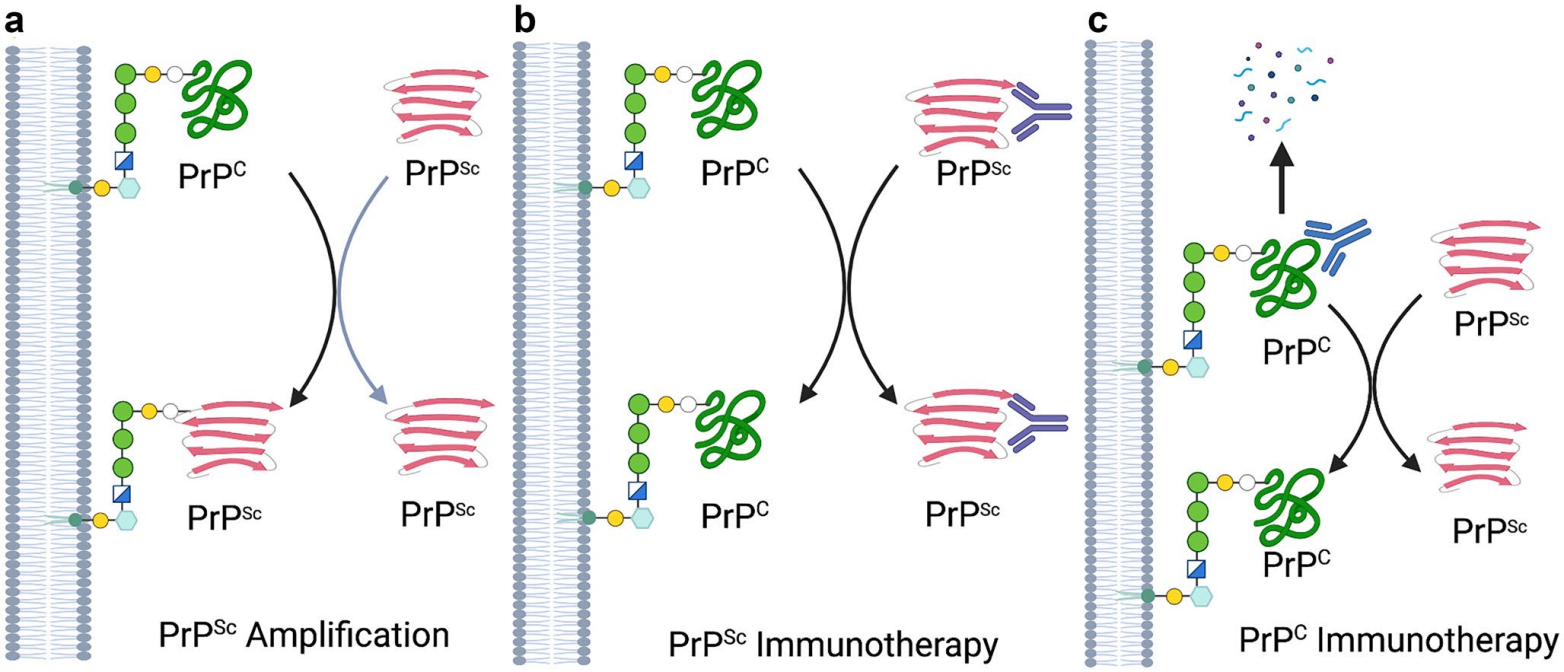
Mechanisms of immunotherapeutic intervention.** A** Natural progression. PrP^Sc^, through physical interaction with PrP^C^, serves as a template for misfolding.** B** PrP^Sc^-specific immunotherapy. Antibodies to PrP^Sc^, through disruption of the interaction between PrP^Sc^ and PrP^C^ block induced misfolding of PrP^C^. **C** PrP^C^-specific immunotherapy. PrP^C^-specific antibodies can block the interaction with PrP^Sc^ as well as causing depletion of PrP^C^

Mechanistically, PrP^C^-targeted immunization aims at induction of self-antibodies that bind at cell surface-located PrP^C^ and thereby remove or impede its conversion into PrP^Sc^ (steric hindrance) (Gilch et al. 2003; Abdelaziz et al. 2017) (Fig. 2). This works well outside the CNS, where self-antibodies do not have to cross the BBB and in situations where prions propagate in the periphery, e.g., in the process of primary infection (CNS invasion) or anterograde transport from the infected CNS to the periphery (prion shedding). To overcome self-tolerance, aggregation-prone and therefore stable recombinant dimeric or monomeric PrP isoforms were used as immunogens. For example, dimeric cervid PrP consists of two cervid PrP moieties (full-length minus signal peptides; aa 23–231), covalently linked by a 7-aa linker (AGAIGGA), and fused to an N-terminal poly-his tag (Gilch et al. 2003; Abdelaziz et al. 2017). The moieties also encode epitope tags and can be used as potential DIVA vaccines. Quality control is done after refolding of protein, assessing quality and purity by gel electrophoresis, immunoblot, size exclusion chromatography, or FTIR. Such examinations showed that PrP dimers are aggregation-prone with more β-sheet structure than monomers have (Kaiser-Schulz et al. 2007). Aggregation can be enhanced when mixed with adjuvant (Kaiser-Schulz et al. 2007). This may result in more stability in vivo and longer exposition to antigen-presenting cells. Recombinant PrP can also be efficiently encapsulated together with adjuvant into biodegradable polylactide-coglycolide (PLGA) nanospheres (Kaiser-Schulz et al. 2007) and such nano-vaccines used for oral immunization.

Overall, there is now a solid proof of evidence that such vaccine candidates can overcome self-tolerance in rodent models and cervids, resulting in detectable humoral and cellular immune responses (Gilch et al. 2003; Kaiser-Schulz et al. 2007; Abdelaziz et al. 2017). Importantly, such targeting of PrP^C^ does not result in adverse side effects (Kaiser-Schulz et al. 2007; Abdelaziz et al. 2017). Results in transgenic mouse models of CWD infection indicate that vaccines can extend prion incubation time up to 60% (Abdelaziz et al. 2017).

### PrP^Sc^-specific antigens

Efforts to focus immune responses to PrP^Sc^ are motivated by dual considerations of safety and efficacy. For safety, given the potential consequences of induction of immune responses to a widely expressed, cell surface protein, there is appeal for conformation-specific immunotherapy. In terms of efficacy, prioritizing the misfolded species could serve to focus the immune response to the most pressing threat (Fig. 2). Conformation-specific immunotherapy is dependent on the identification of epitopes that are specifically exposed for antibody binding in the misfolded state, disease-specific epitopes (DSEs). While conceptually appealing, the identification of these targets is complicated by the tendency of the misfolded PrP to form insoluble aggregates which are unsuitable for biophysical characterization. Despite these challenges, conformation-specific targets, representing both linear and conformational epitopes, have been identified.

### Disease-specific epitopes

The first DSE of PrP was discovered from biophysical investigations of the refolding of PrP^C^ into PrP^Sc^ which revealed unique surface exposure of a YYR-motif. That antisera to this DSE immunoprecipitated PrP^Sc^ from prion-infected brain with an absence of reactivity to PrP^C^ from healthy brains supported the use of the epitope for PrP^Sc^-specific vaccines (Paramithiotis et al. 2003). Initial efforts to further translate this target into a vaccine were hampered by the limited immunogenicity of the tripeptide, but rationale expansion of the core DSE epitope, as well as presentation on a suitable carrier protein, resulted in a vaccine that induced robust PrP^Sc^-specific antibody responses (Hedlin et al. 2010). Parenteral administration of this vaccine delayed onset of scrapie in a sheep challenge model (Taschuk 2014) but accelerated disease onset in an environmental challenge model of elk (Wood 2018). Both challenge models utilized oral routes of infection: within the sheep model, animals were exposed to a large single dose of administered infectious material, while the elk were housed in a prion-infected environment with prolonged exposure to low-level prions. It is uncertain whether the unique outcomes of these trials reflect differences related to the species or the challenge models.

Based on the positioning of the YYR DSE on beta-strand 2 of PrP^C^, it was hypothesized that the opposing beta-strand may undergo similar repositioning to surface exposure upon misfolding. A region of the opposing beta-strand, corresponding to the sequence YML, was confirmed to meet the criteria of a DSE through induction of PrP^Sc^-specific immune responses (Marciniuk et al. 2014). A third DSE was identified through a bioinformatic algorithm that predicts regions of proteins most likely to unfold; the loop region between beta-strand 2 and alpha-helix 2 was implicated, and confirmed, to represent a DSE (Marciniuk et al. 2014). As structural investigations had noted unusual rigidity of this region in cervid PrP (Gossert et al. 2005), this DSE was designated as rigid loop (RL). Through optimization of these core sequences, these DSEs were translated into vaccines that exhibit strong immunogenicity, specificity, and safety profiles when administered individually or in a multivalent format (Marciniuk et al. 2014). Vaccines based on the YML and RL DSEs have not been evaluated for efficacy in animal challenge trials, but their associated antibodies have been shown to neutralize prions in vitro (Taschuk et al. 2015a, b).

### Structurally restrained epitopes

VPrP^Sc^-mimicking surface-exposed structures of PrP^Sc^ are based on a 4-rung beta-solenoid fold of PrP^Sc^ (Wille and Requena 2018), a model recently challenged by others. Seven discontinuous residues surface-exposed in PrP^Sc^ but not in PrP^C^ were selected and inserted into the fungal HET-s prion domain, which adopts a 2-rung beta-solenoid (Wasmer et al. 2008) and is an innocuous and non-pathogenic scaffold, well-suited as a vaccine carrier. Mice injected with VPrP^Sc^ developed an immune response selectively recognizing PrP in prion-infected (but not non-infected) brain homogenates. An transgenic mouse model carrying a mutation in human PrP causing GSS syndrome in humans, immunized with VPrP^Sc^, remained free of clinical signs up to 450 days of age, while unvaccinated controls developed disease at about 177 days (Fleming et al. 2022; Nazor et al. 2005).

### Vaccine formulation and delivery

Most of the efforts to develop prion vaccines have utilized parenteral routes of administration. This an effective approach for investigation of potentially protective epitopes, and an injected vaccine could have application for control of CWD in farmed cervids. However, given the importance of mucosal immunity in protection from oral routes of infection, that mucosal vaccines induce both peripheral and mucosal responses, and that control of CWD in wild cervids will likely depend on the use of oral vaccines; the clear priority is to develop oral prion vaccines. Control of wildlife diseases through oral, self-administered vaccines is an achievable goal, as demonstrated by the highly successful example of rabies (Mahl et al. 2014). Three biological vector platforms have been shown to be effective in inducing mucosal and systemic antibody responses for prion vaccines, including within cervids (Goni et al. 2015, 2008, 2005; Taschuk et al. 2017; Gonzales-Cano et al. 2017).

### Adenovirus vectors

A replication-incompetent human adenovirus serotype 5 (hAd5) was investigated as a platform for an oral CWD vaccine as it has a broad species and tissue tropism, induces systemic and humoral immunity, and can be dosed orally (Buge et al. 1997; Alejo et al. 2013). One of the commercialized oral wildlife vaccines for rabies, OnRab, is based on a human adenovirus platform (Yarosh et al. 1996). Oral delivery of a hAd5 encoding the RL DSE fused to truncated rabies glycoprotein G carrier protein to white-tailed deer-induced PrP^Sc^-specific systemic and mucosal immune responses after two immunizations (Taschuk et al. 2017). The induction of epitope-specific antibody responses confirms the ability of the replication-defective vector to infect cells within the gastrointestinal tract of white-tailed deer. While a replication-competent virus would likely result in higher levels of antigen expression, and therefor elevated immune responses, such a vector presents increased safety risks of environmental contamination and/or unanticipated immunization of non-target species. The hAd5-RL-tgG vaccine showed an encouraging safety profile with no indications of adverse health effects and a limited duration of vector shedding (Taschuk et al. 2017).

### Lambda phage

Bacteriophage have many characteristics consist with delivery platforms for oral vaccines. They are structurally stable in the gastrointestinal tract, are amenable to genetic manipulation, and possess favorable immunogenicity traits. As bacteriophage are ubiquitous within mammalian digestive systems and replicate within bacteria, they are generally regarded as safe to eukaryotic hosts (Hodyra-Stefaniak et al. 2015). To investigate the capacity of phages to induce mucosal immune responses, three prion DSEs were presented as recombinant fusions of the phage capsid head protein D. Following targeted delivery to intestinal segments of calves, the phage particles were taken up from the small intestine into Peyer’s patches to the induction of strong IgA responses to each of the three epitopes, in the absence of a mucosal adjuvant (Gonzales-Cano et al. 2017).

### Bacterial delivery

Live-attenuated strains of *Salmonella enterica* have been used as mucosal vaccines against *Salmonella* infection and as delivery systems for vaccines in human and veterinary medicine (Hegazy and Hensel 2012). The first use of a *Salmonella* delivery system for mucosal vaccination in prion disease was described as early as 2005 (Goni et al. 2005). Such vectors are considered safe, as they are genetically modified in a way to prevent reversion into a disease-inducing state. Goni and colleagues used the *Salmonella enterica* serovar *Typhimurium* strain LVR01 that is attenuated by deleting the gene that encodes for the essential enzyme chorismate synthase. The deleted strain can reach lymphoid follicles in the gut of animals, thereby delivering antigens without any associated virulence (Goni et al. 2008). Interestingly, this vector vaccine expressed tandem copies of PrP, and a higher copy number yielded better results (Goni et al. 2008). It is unclear whether this added only more expression units or whether the expressed PrPs were also tandems at the protein level. This mucosal vaccine was tested in a CWD-infected cervid model, indoor-housed white-tailed deer, for protection against clinical prion disease (Goni et al. 2015) (Fig. 2). Deer received up to eight immunizations using different routes, mainly oral exposition by gavage, to *Salmonella* vaccine with alum as adjuvant. The immunized group showed a significant prolongation of incubation time compared to the control group, with one out of 5 animals not showing signs of infection after 3 years (Goni et al. 2015). This animal also had high anti-PrP IgA titers in saliva and IgG in blood. Overall, this provides a proof-of-concept that oral vaccination can provide protection against CWD infection in cervids.

## Evaluating prion vaccines

### Available animal models

Evaluation of prion vaccines depends strictly on appropriate in vivo models. Whereas immunological parameters and side effects of vaccination can be evaluated in uninfected animals, determination and quantification of protection from infection require animal infection, ideally within a target species and with characteristics of infection that most closely mimic real-world transmission in terms of the prion strains, dose, and routes of infection. Consequently, while prion vaccines intended for ungulates should be tested in the homologous large animal model, this is often not feasible and rodent models can provide viable alternatives.

Mouse-adapted sheep prion strains replicate well in wild-type mice and provided a first animal platform to study prion vaccines. Prions from ungulates and humans often do not replicate well in non-transgenic mice and hamsters, and the more broadly susceptible bank voles were not studied so far for vaccine purposes. On the other hand, there are transgenic mouse models that overexpress bovine, ovine, cervid, or human PrP, which replicate prions of the respective species and develop a clinical prion disease with known and predictable incubation times (Buschmann et al. 2000; Vilotte et al. 2001; Browning et al. 2004; Wadsworth et al. 2010). The disadvantage is that overexpressing transgenic mice usually do not fully recapitulate prion biogenesis and pathology as found in the source animal. For example, they cannot be infected using oral or intraperitoneal routes or do not provide a 100% attack rate, making it difficult to compare vaccinated with non-vaccinated control groups (Seelig et al. 2010; Abdelaziz et al. 2017). Very often, they also do not shed prions like a natural host; a prime example are transgenic mouse models for CWD.

Knock-in (KI) mice can overcome these important drawbacks of overexpressing transgenic mice. They express the PrP transgene under the authentic PrP promoter, with correct spatial and temporal expression levels. For cervidized mice, two groups have produced such mice, encoding various cervid genotypes (e.g., deer, elk, and caribou/reindeer) (Bian et al. 2019; Arifin et al. 2022). As expected, such mice develop clinical prion disease with a 100% attack rate upon oral and intraperitoneal inoculation, with incubation times only slightly longer than for intracerebral (i.c.) infection. KI mice come down with disease later than transgenic mice upon i.c. inoculation, as they do not overexpress PrP. Importantly, they seem to fully recapitulate CWD pathogenesis and prion lateralization as found in the cervid host, which includes shedding of CWD prions into feces, saliva, and urine (Bian et al. 2019). This allows usage of cervid PrP KI mice to study vaccine effects on CWD prion shedding, which was not possible to do in existing transgenic mice.

### Mouse versus large animal models

Whereas rodent models have the advantage of feasibility, including large number study sizes (and both sexes), appropriate biosafety and well-controlled, and reproducible experimental conditions, they are still a “model.” The gold standard to study vaccine efficacy for ungulate prions would be the respective large animal, similar as an animal model cannot substitute for phase 2 and 3 trials in humans. In ruminants, the gastrointestinal tract is very different from the one in rodents, which is relevant for vaccine stability upon oral delivery as well as various immunological parameters. In addition, what we know from mouse and human immunology can be different for innate and adaptive immunity in ungulates. For example, the proportions of non-conventional B and T cell populations, which can impact reactivity to conformational versus linear epitopes, non-peptide-based antigens, and immunological memory, are different and less well studied.

For experimental studies in cervids, there are two basic scenarios. One is controlled oral challenge studies in cervid species kept inside, for example, WTD and reindeer (Mathiason et al. 2006; Mathiason 2022; Mitchell et al. 2012). Similar studies have been done with outdoor-held captive elk in appropriate confinements (Basu et al. 2012). Each individual animal receives a well-defined oral dose of characterized CWD prions, usually via gavage and under anesthesia. This results in a 100% attack rate, with predictable incubation time to clinical disease, which facilitates appropriate sampling of biological materials (e.g., lymph node biopsies, saliva, urine and feces) (Haley et al. 2009, 2011; Henderson et al. 2013). Since a vaccinated population is compared to controls only receiving adjuvant, such studies allow assessing vaccine efficacy with regard to protection, mitigation, and prion reduction in biological materials (Goni et al. 2015). The time frame for such costly studies is in the 2–3 years range at minimum and can only be performed in a handful of facilities worldwide. The alternative model uses captive or even free-ranging cervids, vaccinates, and exposes them/keeps them exposed to CWD in the environment, with controls not receiving vaccination. This can be pastures of recently depopulated farms or areas with high CWD prevalence in free-ranging animals in an environmental setting. When using free-ranging cervids, some animals likely are already inoculated and in various stages of infection. The environmental exposure mimics naturally occurring CWD infection, likely smaller doses over longer periods, and is therefore closer to the real-life scenario (Taschuk et al. 2017; Wood et al. 2018). The drawback is that it remains unknown whether, when and how much CWD inoculum is taken up. Therefore, incubation time and attack rate cannot be predicted with accuracy, and additional animal numbers are necessary to obtain statistically significant differences between vaccinated and unvaccinated groups. There might also be regulatory challenges, and a certain level of confinement is necessary, to exclude release of vaccines to unvaccinated animals or other species. Since the challenge with CWD is an uncontrolled spreading of CWD in wild, free-ranging animals, e.g., deer, elk, and soon caribou in North America, there will be no way around such a real-world scenario with appropriate oral delivery, which might need to be tailored towards a given species.

### Routes of prion infection

When inoculating animals in experimental prion research, the gold standard is using the intracerebral (i.c.) route, because it usually provides the highest attack rates and the shortest incubation times to clinical prion disease (Scott et al. 1989; Weissmann et al. 1996). Whether this is the most appropriate route of infection for prion vaccine studies depends on the objectives of the investigation. In instances where the objective to is evaluate protective effects of vaccination in the CNS, i.c. infection is appropriate. In other scenarios, vaccination targets early events in peripheral prion infection and/or extra-CNS prion propagation, and therefore, infection is better performed by either intraperitioneal (i.p.) or oral routes. The first recapitulates the process of neuroinvasion and the roles the lymphatic and peripheral nervous systems play. The latter addresses mucosal immunity, and the potential interplay between prions taken up orally and Peyer’s patches as part of the mucosal-associated lymphatic tissue (MALT), M cells, and secretory IgA, for example. Cervids, sheep, and cattle are ruminants, and how orally ingested prions must pass their digestive tract and when innate and adaptive immunity could come into play is very different from the situation in experimentally infected rodent models. Most importantly, wildlife vaccination of cervids will need an effective oral vaccine strategy, so oral vaccination in the natural host must be the goal.

### Parameters to assess vaccine success

Given the time, expense, and difficulties associated with prion challenge trials, it is often useful to have immunological markers of vaccine-induced responses that can be used to prioritize and optimize vaccine candidates. On the immunological side, typically humoral responses are studied, which can be done in the absence of infection. Antibodies reacting with PrP (PrP^C^ or PrP^Sc^) are usually tested in ELISA or immunoblot, in quantitative and qualitative formats. For ELISA, recombinant PrP or purified PrP^Sc^ can be used as antigen, keeping in mind whether the PrP conformation is native, renatured, or denatured. An intra-individual increase of specific reactivity (above cut-off) compared to pre-immune sera is measured, and it is important to include also adjuvant-only treated controls (Gilch et al. 2003; Taschuk et al. 2017; Abdelaziz et al. 2017). The ability to bind to native PrP can also be quantified by flow cytometry using PrP^C^-expressing cells (Polymenidou et al. 2004). Since reliable detection of PrP^Sc^ on the outer leaflet of the cell membrane of prion-infected cells is still a matter of debate, this approach is not established for assessing antibodies binding to native PrP^Sc^. Reactivity to linear epitopes is mapped in peptide-based ELISA assays. Such reactivity does not, however, indicate whether the anti-PrP antibodies are “protective,” like neutralization assays in virology, the prion infection-neutralizing capacity can be studied in cell culture and in vitro prion conversion assays (Gilch et al. 2003; Abdelaziz et al. 2017).

## In vitro neutralization assays

### RT-QuIC interference assay

Since this assay allows to discriminate substrate (recombinant PrP) and template (PrP^Sc^ seed) (Wilham et al. 2010; Orrù et al. 2012; Haley et al. 2018), it is almost ideal to dissect whether humoral immune responses are directed against PrP^C^, PrP^Sc^, or both. This can be achieved by simply pre-incubating either of the two components with a dilution series of the post-immune antisera (Abdelaziz et al. 2017). Quadruplicate reactions are analyzed on plate readers by measuring ThT fluorescence increase. RT-QuIC data are plotted against reaction time and scored positive if at least 50% of replicates reach the fluorescence cut-off. In a convenient 3-day format, this assay demonstrates if antisera can partially sequester prions in the prion seed and decrease conversion activity or if they block the PrP substrate.

### Cell culture neutralization assay

This assay studies the effect of anti-PrP antibodies in the context of a cellular prion infection and usually uses persistently prion-infected cell lines (Butler et al. 1988; Schätzl et al. 1997; Mahal et al. 2007). Typically, cells are cultured for several days in medium with diluted serum of vaccinated animals, using pre-immune sera as a control. Aliquots of cells are lysed or further passaged in the presence or absence of antibodies. This assay is complex, can take several weeks when cells are passaged, and is mostly semi-quantitative as changes in PrP^Sc^ levels are assayed in immunoblot (Gilch et al. 2003; Abdelaziz et al. 2017). Results demonstrate whether the vaccine-induced antibodies can disrupt cellular prion propagation. Alternatively, a more standardized scrapie cell assay (SCA) can be employed that tests new infection of susceptible cells (Klöhn et al. 2003; Bian et al. 2010). The assay is very sensitive, has a short turn-around time (days), and is quantitative. Similar to RT-QuIC, prion inoculum and recipient PrP^C^-expressing cells can be separately pre-incubated, dissecting reactivity towards the different isoforms. Available cells can propagate mouse-adapted, scrapie, and cervid prions, which limits applicability. T cell responses are less often analyzed, using, e.g., classical flow cytometry-based assays for intracellular cytokine staining or EliSPOT-based assays. It is likely that in future studies, single-cell RNASeq analysis will play a prominent role to address the quality of immune responses.

To address the protective effect of prion vaccination on a whole-animal level, animal models that can be infected with the respective prions are necessary. Usually, animals are vaccinated followed by prion challenge, and experimental modalities were described above. Incubation time to clinical prion disease is the main read-out. A relative effect would be some prolongation; full protection would mean vaccinated animals do not develop a clinical prion disease. Additional readouts are levels of PrP^Sc^ in certain tissues are body fluids, e.g., lymph nodes or saliva, which can be done intra vitam (Haley et al. 2009, 2011; Henderson et al. 2013). Besides prion lateralization in peripheral tissues, the effects of vaccination on shedding of prions into feces and urine is another important read-out, which can be done by using ultra-sensitive prion conversion assays like PMCA and RT-QuIC.

Finally, it is important to exclude unwanted side effects, by clinical observation and/or lab testing. Whereas lymph nodes can be assessed by biopsies, more detailed histopathological analysis needs euthanasia of the animal (Kaiser-Schulz et al. 2007). The spectrum of side effects can range from signs of acute or hyper-acute inflammation to severe pathologies in tissues like the kidney and brain (e.g., autoimmune-encephalitis). For the latter, it plays a role whether induced antibodies are supposed to cross the blood–brain barrier and penetrate effectively into the CNS. Another problem could be the induction of antibody-dependent enhancement (ADE), which was observed for several viral vaccines (Halstead et al. 2010). In this situation, vaccination does not reduce the infection process but instead enhances it. For the prion diseases, this could potentially occur through the antibodies promoting the misfolding of PrP into an infectious conformation. Alternatively, the presence of PrP^Sc^-reactive IgA antibodies in the mucosal surfaces of the intestine could serve to increase uptake of ingested prions. Another potential mechanism of vaccine enhanced prion disease could be by non-neutralizing antibody decoration of the agent, with improved uptake by certain immune cells in a Trojan horse mechanism. Since the role of macrophages is not fully clarified in prion infection, such a possibility needs attention. Finally, there is the possibility that vaccine-induced responses could result in the trafficking of prions to lymphoid tissues which offer a more permissive environment for prion amplification, thereby accelerating disease (Beringue et al. 2012). In summary, with the potential for a variety of negative effects with prion vaccination, researchers must place high priority on evaluation of the safety of all vaccine candidates.

## What does success look like?

### Is there a universal prion vaccine?

Since prions strictly use endogenous PrP^C^ of the host for their propagation, prions generated in humans, ungulates, and rodents differ in their primary sequence. For example, ruminant PrPs differ by ~ 8% compared to human PrP at the amino acid level and rodent PrPs by up to 15% (Schatzl et al. 1995). Although the overall identity seems high for PrP^C^, there could be important structural differences in the PrP^Sc^ scaffold that might affect immunological outcomes. Whether the prion disease is sporadic, genetic, or acquired by infection may impact the success of vaccination efforts as origins in either the periphery or CNS will impact the accessibility of the prions to the immune system. Prima vista, acquired prion diseases appear more prone to vaccine success, but it has to be seen whether this assumption holds true.

For vaccines targeting PrP^Sc^, a universal vaccine depends on how “conserved” disease-specific or structurally restrained epitopes are. While the core epitopes of the three prion DSEs characterized to date are highly conserved across species, the expansions of these regions to improve immunogenicity often introduce species-specific sequence variations that could potentially impact the ability of the induced antibodies to react with PrP^Sc^ molecules from different species or even naturally occurring polymorphisms from within the same species. This would need to be investigated and potentially accounted for through multivalent vaccines that either target multiple DSEs or multiple sequence variants of the same DSE.

For vaccines targeting PrP^C^, this is not unlikely, as PrP^C^ is a protein highly conserved within mammals (Schatzl et al. 1995; Wopfner et al. 1999). In addition, since a conserved cellular protein is targeted, there is no immune evasion to expect. Apart from the design of the immunogen itself, delivery and route of application of vaccines will play a role. For example, free-ranging cervids will need an oral vaccine strategy, maybe delivered by a vector, whereas vaccination of farmed cervids can be done by injection. Oral vaccines in cervids will likely need delivery by baits, and MD, WTD, elk, or caribou has different needs in this respect. Taken together, although a prion vaccine likely will have some “universal” character, its packaging and delivery must be optimized for the target population.

### Impact of prion strains

Another variable which might affect vaccine success is the existence of prion strains (Bessen and Marsh 1992; Carlson et al. 1994; Bessen et al. 1995; Weissmann 2009). Within a given species, there can be a variety of prion strains that differ in conformation and biophysical properties (Collinge 2010; Carta and Aguzzi 2022). Strains are not static; they can change, evolve, and undergo selection under pressure (Li et al. 2010; Wadsworth and Collinge 2011). This dynamic scenario is further complicated by extensive genotypic variability in certain species, known as PrP polymorphisms (Goldmann 2008; Arifin et al. 2021). The proposed quasi-species nature of prion strains might affect vaccine success and predispose, at least in theory, to the development of immune escape populations over time, solely based on selection of protein conformations (Li et al. 2010; Wadsworth and Collinge 2011). This is a well-known scenario for viral diseases, here based on mutations on the nucleic acid level, and the failed vaccine attempts in HIV-1 are an extreme example. Another possibility is the before mentioned ADE, based on suboptimal antibody affinities. Univalent vaccines based on minimal epitopes could potentially be at higher risk for loss of efficacy in the face of escape variants, although the extent of structurally variability of prion strains impacts reactivity with such antibodies has yet to be determined. Vaccines targeting PrP^C^ may have greater opportunity to maintain efficacy in the face of evolving strains and may be less predisposed to such resistance or ADE scenarios.

### Absolute protection vs slowing of disease/reduced shedding

Given the 100% fatal nature of prion diseases and the suboptimal recognition of prions by the host immune system, sterilizing immunity from prion infection is likely an unrealistic goal. From what we have seen in experimental vaccination studies published so far, it is more likely that protection is relative, translating into a prolongation of incubation time (Polymenidou et al. 2004; Goni et al. 2015; Taschuk et al. 2017; Abdelaziz et al. 2017). Nevertheless, these results are very encouraging, and active vaccination could be a cornerstone in the attempts to contain CWD, both in wild-living and farmed cervid populations. Slowing down CWD development would create less casualties, take away pressure from populations, and allow them to cope and come up with countermeasures (one is starting the reproduction age earlier). This would also positively affect food security in regions depending heavily on hunted cervids. As a note of caution, an increase in lifetime could result in dissemination of more infectivity into the environment over time, if the vaccine does not also reduce CWD shedding. This is reminiscent of similar examples in veterinary medicine, where vaccines do not eliminate the agent in vaccinated animals, resulting in persistently infected and chronic active carriers. A CWD vaccine that also reduces CWD prion shedding would reduce the load of prions in the environment over time, result in less infections in the future, and thereby also reduce the zoonotic risk. Taken together, a vaccine that combines increased survival, better population fitness and reduced shedding of CWD infectivity will be a great success and a key element in successful CWD management.

### Vaccines for animal prion diseases

CWD is currently the most pressing prion disease of animals. Within that, both farmed and wild cervids are impacted, and vaccines for each of those populations faces unique technical requirements as well as different standards of “what success looks like.” For farmed cervids, there is greater opportunity to control vaccination parameters to achieve a protective response. This includes the option for either oral or injected vaccines, as well as control over the number and timing of vaccinations. In contrast, vaccination of wild cervids is limited to oral vaccines with minimal control to ensure that all animals are vaccinated, the dose of vaccine received by each animal, and uncertainty over whether animals receive booster immunizations. Vaccines released into the environment also face conditions that could compromise their efficacy. With this, there is greater requirement for wildlife vaccines to be durable, with the induction of long-lasting immune responses, even after a single vaccination.

While prion vaccines for farmed cervids face less stringent technical requirements, they also face a higher standard of success. For farmed cervids, particularly those utilized as sources of food or alternative medical products, there is priority to ensure the health of individual animals. This reflects the inability of infected animals to recover from disease, as well as resistance of prions to treatments that neutralize the infectious agent without compromising the quality of the associated animal product. Should CWD ever be confirmed as a zoonotic disease, as emerging evidence suggests, these standards are certain to become even more stringent. In contrast, for wild cervids, the priority is to protect populations rather than individual animals. This can theoretically be achieved by reducing the amount of infectious material that is generated and spread by infected animals. The reduced burden of prions in the environment, in combination with other disease control measures, will hopefully break the cycle of disease transmission and eventually reducing CWD to manageable levels.

### Vaccines for human prion diseases

Sporadic CJD, the main form in humans, is a very rare disease without preclinical biomarkers (Hermann et al. 2021). To protect humans from sporadic CJD mass vaccinations would be required, which is not realistic given the low disease prevalence. When clinical symptoms are already present in a patient, vaccination most likely would be too late. In addition, such a vaccine would have to exert its protective effect in the CNS. Human prion diseases acquired by infection, for example, vCJD or iatrogenic CJD, would have similar challenges as sporadic CJD and not provide a valid medical indication for vaccination. Certain post-exposure situations might be different, as here the potential time point of infection is known, and given the very slow process of neuroinvasion until establishment of infection in the CNS, vaccination could exert positive effects. A potential example could be a laboratory accident with human prions or potentially zoonotic prions.

The situation for genetic prion diseases, which includes familial CJD, GSS, and familial FFI, might represent opportunities for vaccination. These diseases are inherited within families and can be diagnosed by simple DNA analysis, and incubation times to clinical disease are roughly known, although with great inter-individual variations. Vaccination could start long before clinical manifestations, with administration of necessary vaccine boosters over time, and laboratory testing for induced immune responses as well as careful monitoring for potential side effects. In fact, experimental vaccine candidates based on structurally restrained epitopes have been tested in two transgenic mouse models of GSS, showing impressive prolongations of survival time (Fleming et al. 2022). This looks very promising, and this approach might even work for other familial neurodegenerative diseases that involve prion-like mechanisms, for example, AD and PD.

### Potential impacts of a vaccine for control of CWD

**Fig. 3:**
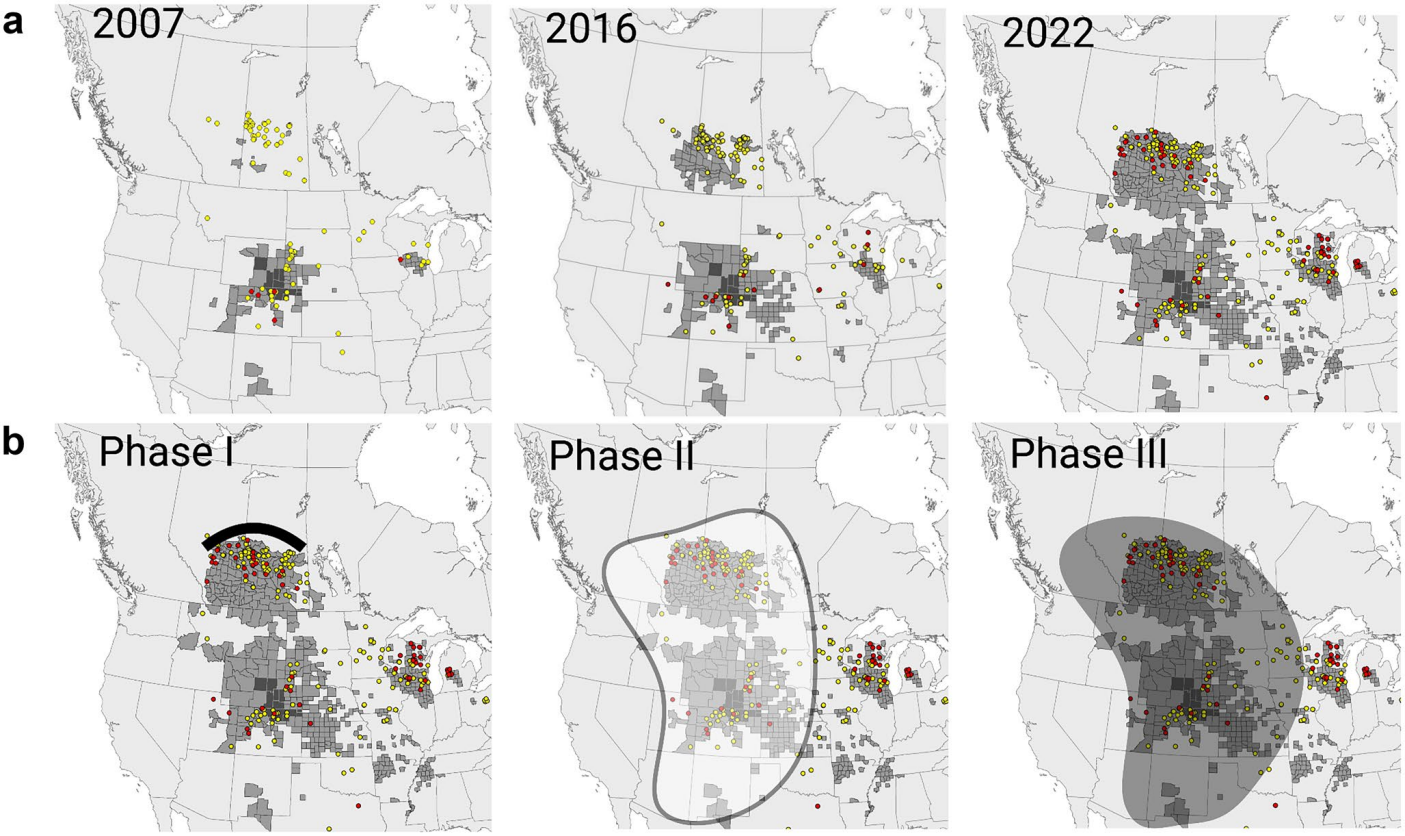
Progression of CWD and potential deployment strategies of CWD vaccines.** A** Incidence of CWD over the past 15 years.** B** Strategies for control of CWD through vaccination. Phase I. Strategic deployment of oral vaccines in the wild to limit the spread of disease into critical regions such as the northern regions home to caribou. Vaccination of farmed cervids with either oral or injected vaccine. Phase II. Establish complete vaccination rings around regions where CWD is endemic. Phase III. Distribution of oral vaccines within vaccination rings to reduce incidence of disease. Maps are adapted from the US Geographical Survey (USGS)

It is important not to underestimate the threat presented by CWD nor the challenges to its effective management. This includes the widespread geographic occurrence, CWD has been spreading aggressively through North America (Fig. 3A), environmental persistence of the agent, the unique molecular mechanism of propagation, occurrence in wildlife species, and evolving nature of the threat. With that, it is important to set realistic expectations of the extent and time frames in which the trajectory of the disease can be impacted. While an effective oral vaccine would represent a critical resource, the challenges of CWD are likely too great to rely on a single tool for disease control. Instead, vaccines are envisioned as a component of a multi-pronged approach that could include strategic culling, understanding/influencing genetic susceptibilities to infection, and neutralization of environmental prions. Within those efforts, vaccines will likely contribute within two realms. Firstly, by reducing the quantity of prions generated within an infected animal, it may be possible to begin to reverse the trend of the increasing environmental burden of prions. Given the extended survival of environmental prions, this will be a slow process. The second area where vaccines could serve to manage prion disease is through limiting the geographical spread. Given the gradual, predictable, pattern of migration of the disease, the priority might be strategic vaccination in regions where the disease is feared to spread, for example, limiting the spread into northern regions where it may threaten caribou populations (Fig. 3B). A second phase of vaccine distribution could involve attempting to establish rings of vaccination around regions where CWD is already endemic in effort to limit further spread of the disease. As a third phase, the vaccine could then be distributed within these endemic regions to minimize new cases and minimize shedding from existing cases. Once these phases are complete, it is likely that prolonged and ongoing distribution of the vaccine will be required to achieve control of the disease. The timing, location, and intensiveness of vaccine release being informed by characteristics of vaccine in terms of environmental durability and duration of the induced response, consideration for wildlife specialists who can provide information of the timing of patterns of animal movement, and contributions of disease modeling to monitor the impacts of the disease and further define parameters of vaccine utilization to achieve the determined goals.

## Conclusions

The field of vaccinology is pushing into new frontiers of vaccine development and application. This includes rapid acceleration of vaccine technologies, like mRNA vaccines, to deal with the COVID-19 pandemic as well as the development of vaccines for new realms of application like cancer or drug addiction. There is optimism that we have only begun to actualize the potential of vaccines. This includes encouraging progress into the development of vaccines for prion and prion-like diseases. Given the tremendous toll of these diseases on human and animal health, it is critical that we build on these successes to develop essential new tools for these diseases.
